# Two Surgical Cases of Colorectal Cancer in Patients With Immunoglobulin G4-Related Disease

**DOI:** 10.7759/cureus.57267

**Published:** 2024-03-30

**Authors:** Makoto Takahashi, Kazuhiro Sakamoto, Takuo Hayashi, Hisashi Ro, Kiichi Sugimoto

**Affiliations:** 1 Department of Coloproctological Surgery, Juntendo University Faculty of Medicine, Tokyo, JPN; 2 Department of Human Pathology, Juntendo University Faculty of Medicine, Tokyo, JPN

**Keywords:** igg4-related disease, pet/ct, immunoglobulin g4, surgery, lymphadenopathy, colorectal cancer

## Abstract

Immunoglobulin G4-related disease (IgG4RD) is a relatively new disease concept that is most common in Asia. It is a systemic chronic lymphoproliferative disease that is diagnosed by mass formation or thickened lesion, a high serum IgG4 level (≥135 mg/dL), and confirmation of lymphocytes and plasma cells by histopathological examination. The precise mechanism of this disease remains elusive; however, distinguishing IgG4RD from malignancy proves challenging due to its manifestation of swollen lymph nodes and retroperitoneal thickening and fibrosis. Malignancy is also 3.5 times more likely in cases with IgG4RD. In this study, we report two cases of colorectal cancer in patients with IgG4RD who underwent surgery. In both cases, excising the tumor from the retroperitoneal posed a challenge, and swollen lymph nodes were observed without evidence of cancer metastasis. We believe that these are very informative cases, and we report the cases with a literature review of IgG4RD.

## Introduction

Immunoglobulin G4-related disease (IgG4RD) is a chronic lymphoproliferative immune system disorder characterized by high levels of serum IgG4 and fibrotic, tumor, or thickened lesions in various organs, such as the central nervous system, lacrimal gland, salivary gland, thyroid gland, lung, pancreas, bile duct, liver, digestive tract, kidney, prostate, retroperitoneal space, lymph node, artery, skin, and breast [1]. IgG4RD is a relatively new disease concept, for which comprehensive diagnostic criteria were established in 2011 and consisted specifically of the following three components: clinical confirmation of growth, mass, nodule, or thickened lesion; a high serum IgG4 level (≥135 mg/dL); and confirmation of lymphocytes and IgG4-positive plasma cells by histopathological examination [1]. Because the disease is associated with lymphadenopathy and retroperitoneal fibrosis, even in the absence of malignancy, it is important to differentiate IgG4RD from malignancy. Patients with IgG4RD are known to be susceptible to malignancy [2,3]. Here, we report two surgical cases of colon cancer with IgG4RD, which we believe will be interesting and useful. This is because, particularly for surgeons, the presence of IgG4RD during surgery may pose a greater challenge in organ dissection than usual, necessitating more careful dissection techniques. Furthermore, preparation for open surgery may be necessary in addition to minimally invasive procedures. Informed consent was obtained from each patient for all published information, and the anonymity of the patients has been maintained.

## Case presentation

Case 1

A 60-year-old woman visited another hospital nine years ago due to a fracture of her right leg. During a blood test at that time, total protein levels were elevated and albumin was decreased. In protein electrophoresis and urine analysis, serum IgG4 was found to be elevated, with high levels of IgG and β2-microglobulin. A computed tomography (CT) of the chest, abdomen, and pelvis was performed due to the high serum IgG4 level and low-grade fever. Since CT revealed swollen mediastinal lymph nodes, a tissue biopsy was conducted by the department of thoracic surgery, considering the differential diagnosis of this lesion, such as IgG4RD, Castleman disease, sarcoidosis, malignant lymphoma, and lung cancer (details of the procedure are unavailable). A pathological examination indicated IgG4+ multisystemic organ lymphoproliferative syndrome (MOLPS), leading to a final diagnosis of IgG4RD. Treatment with 40 mg of prednisolone per day was initiated, and the fever rapidly improved. Subsequently, the patient was observed as an outpatient, and prednisolone was gradually tapered to a maintenance dose of 5 mg daily.

**Figure 1:**
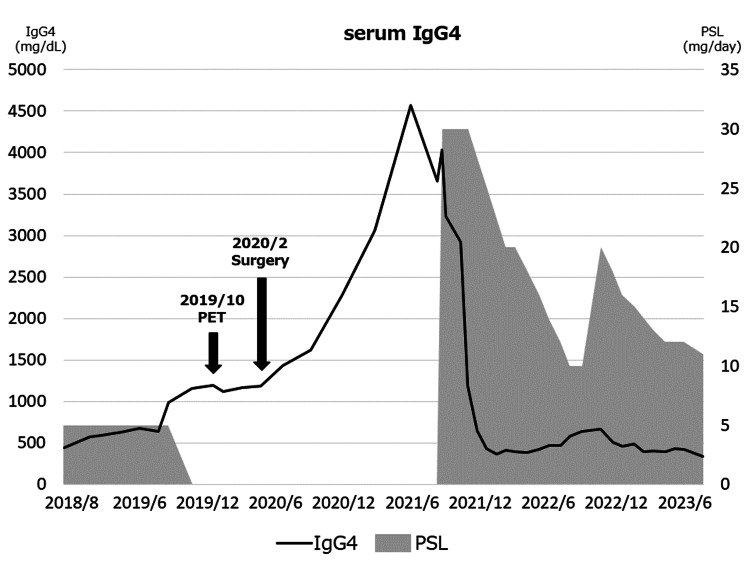
Course of serum IgG4 and PSL treatment IgG4 was elevated preoperatively and then rose further, rather than declining, postoperatively, leading to the resumption of steroid therapy. IgG4: immunoglobulin G4; PSL: prednisolone

The patient moved closer to our institution and presented to our hospital for a follow-up on her disease. Follow-up of IgG4RD began in September 2018. The first serum IgG4 measurement at our hospital was 441 mg/dL (normal range: 11-121 mg/dL) in September 2018. As the overall condition stabilized, prednisolone was discontinued in September 2019. However, serum IgG4 suddenly elevated to 1160 mg/dL (Figure 1)

**Figure 2:**
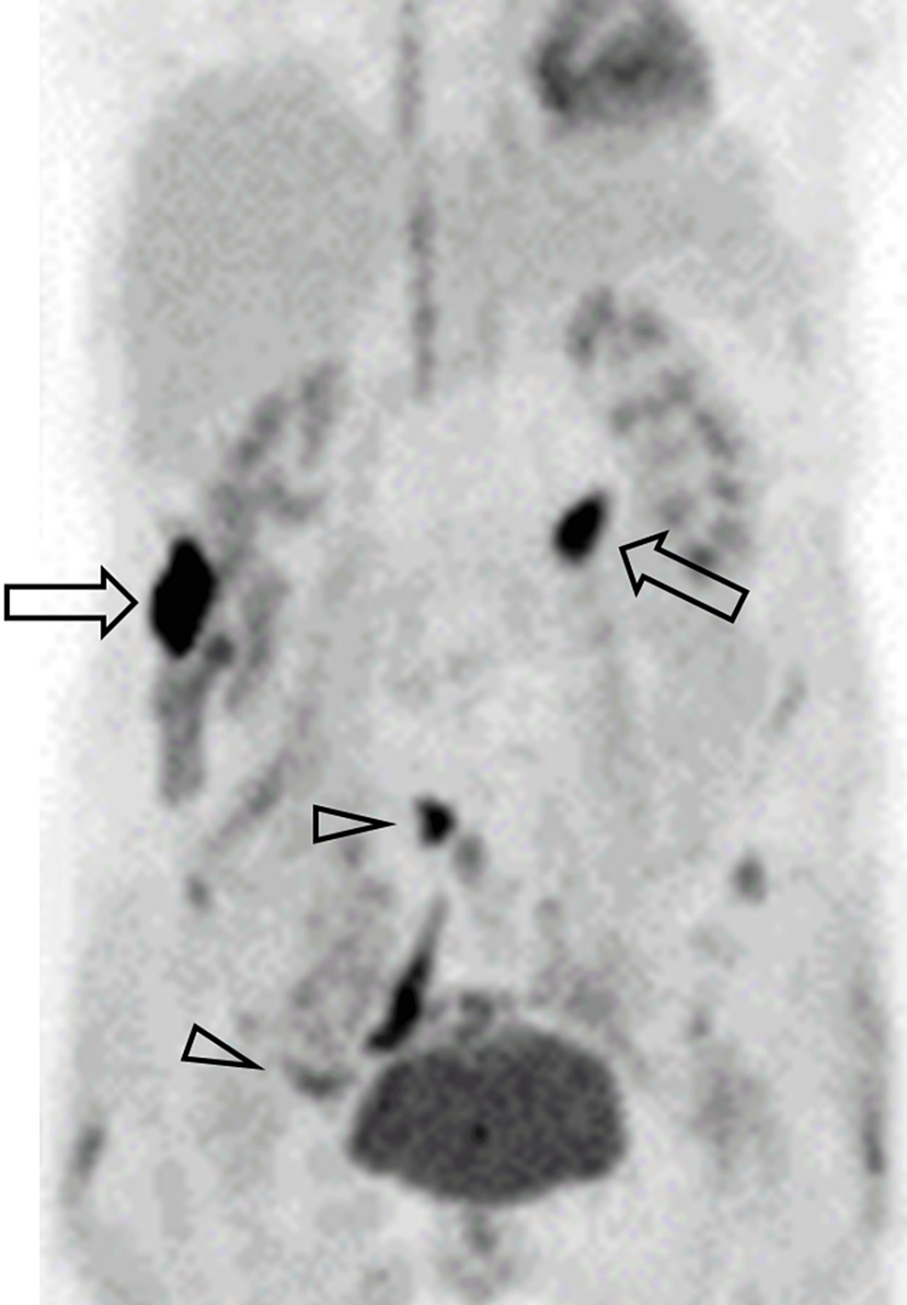
PET/CT PET/CT shows two positive spots in the ascending colon and transverse colon (arrows) and light positive spots in the pelvic area (arrowheads). PET/CT: positron emission tomography/computed tomography

Because IgG4RD is known to induce inflammation in various organs throughout the body [4], positron emission tomography (PET/CT) was conducted due to its superior performance in assessing the activity of IgG4RD and monitoring treatment effects [5,6]. PET/CT showed many nodules with high fluorodeoxyglucose uptake in the ascending and transverse colon and retroperitoneum (Figure 2).

**Figure 3:**
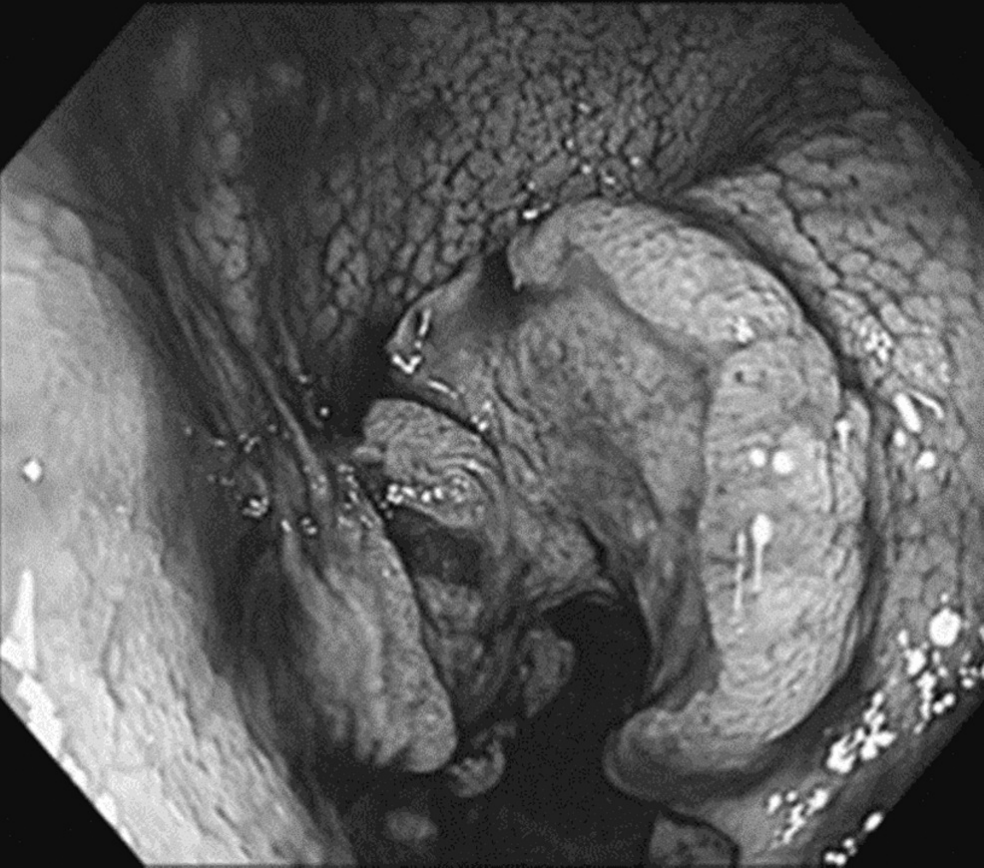
Colonoscopy Colonoscopy shows a one-third circumferential ulcerative tumor in the transverse colon.

**Figure 4:**
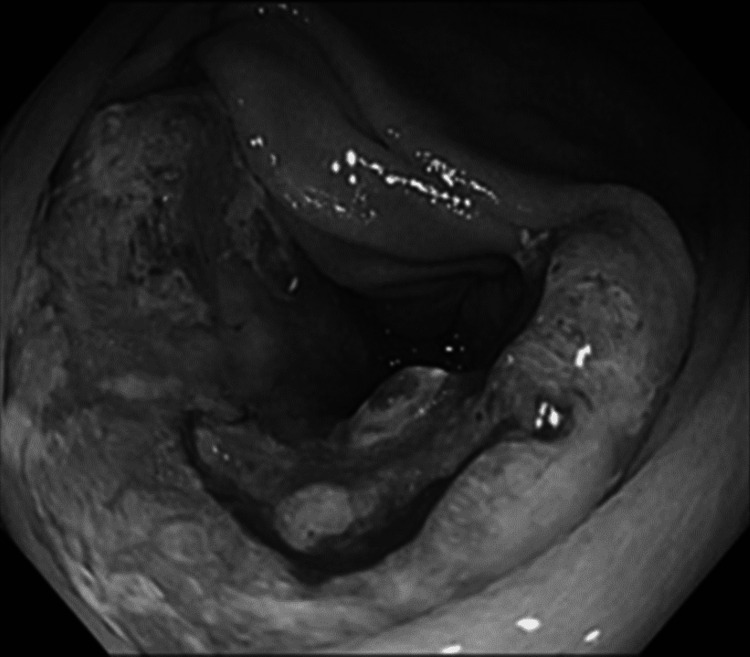
Colonoscopy Colonoscopy shows a two-thirds circumferential ulcerative tumor in the ascending colon.

Colonoscopy revealed a one-third circumferential ulcerative tumor located slightly distal to the anus in the middle of the transverse colon (Figure 3) and a two-thirds circumferential ulcerative tumor in the middle of the ascending colon (Figure 4), both allowing passage of the endoscope.

**Figure 5:**
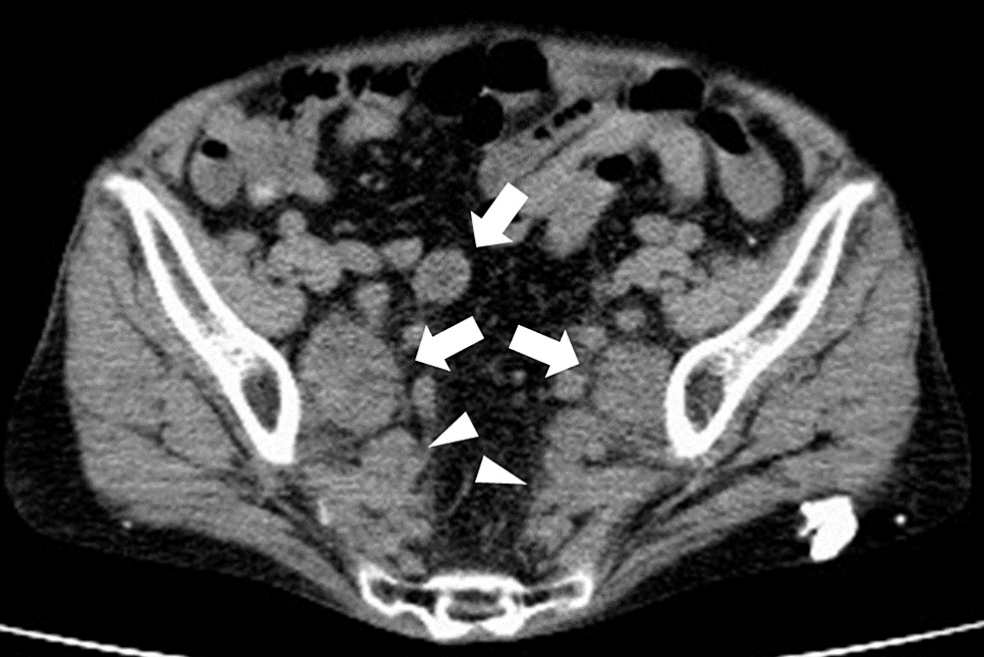
CT CT indicates multiple swollen lymph nodes (arrows) and a thickened retroperitoneum (arrowheads). CT: computed tomography

Biopsies were taken from both tumors and histologically, they were both identified as moderately differentiated adenocarcinomas. Plain CT showed no distant metastasis, but there were many swollen lymph nodes and a thickened retroperitoneum (Figure 5).

The enlarged pelvic lymph nodes and thickened retroperitoneal lesions did not show significant changes compared to previous CT scans. Based on these findings, the preoperative diagnosis was synchronous multiple primary lesions of ascending and transverse colon cancer (T4aN1aM0 Stage ⅢB /T3N0M0 Stage ⅡA) [7] and swollen lymph nodes and thickened lesions due to IgG4RD. A laparoscopic extended right hemicolectomy was performed. The right-side colon was removed; however, fibrosis was stronger than usual, and dissection was difficult. However, finally, we were able to secure a 10 cm anal-side margin of the transverse colon lesion with 10 cm of the ileum, extract the specimen, and create an anastomosis between the transverse colon and ileum without removing or resecting the descending colon. The operative duration was 354 minutes, and the blood loss was 20 ml.

A postoperative histopathological examination indicated ascending colon cancer: subserosa (SS), moderately differentiated adenocarcinoma, pN0 (0/15); and transverse colon cancer: submucosa, moderately differentiated adenocarcinoma, pN1a (1/27). Severe chronic inflammatory cell infiltration was also observed in the specimen. The final diagnosis was T3N0M0 Stage ⅡA and T1bN1aM0 Stage ⅢA, respectively. Postoperatively, adjuvant chemotherapy with a combination of oral uracil-tegafur plus leucovorin was administered for six months. One year after the surgery, a CT-guided needle biopsy was performed on the thickened retroperitoneum in the pelvic cavity to determine whether the lesion was associated with IgG4RD. Fibrosis with lymphoplasmacytic infiltration was observed. Immunohistochemical staining revealed 35 IgG4-positive cells/HPF with an IgG4/IgG ratio >40%. However, there was no clear evidence of storiform fibrosis and obliterative phlebitis, and although the pathological findings did not entirely fulfill the diagnostic criteria for IgG4RD, the possibility of IgG4RD was suggested. There has been no recurrence in about three years since surgery. Immediately after surgery, the serum IgG4 level did not fall; however, after administration of steroids for worsening IgG4RD, serum IgG4 began to fall quickly and eventually reached a lower level than that before surgery, which is higher than the standard value (Figure 1).

Case 2

A 70-year-old woman had been diagnosed with IgG4RD with retroperitoneal thickening without any symptoms and a high IgG4 level in 2000 and had been under observation without any medications. The oldest serum IgG4 value that could be confirmed was 683 mg/dL in 2010. The patient was subsequently followed up regularly by the department of internal medicine. The serum IgG4 level in October 2019 was 442 mg/dL. A few months later, a colonoscopy was performed because a regular health check-up revealed a positive fecal occult blood test. Cecal cancer was found, and CT showed marked thickening of the retroperitoneum in the pelvis. No distant metastasis was observed. Based on these findings, we diagnosed cecal cancer (T3N1aM0 Stage ⅢB) [7].

Ileocecal resection was performed, with the procedure started laparoscopically; however, due to difficulty in laparoscopic manipulation caused by the dilation of the small intestine, which was presumed to be due to cecal cancer obstruction, the approach was converted to open surgery. During the process of removing the right-sided colon from the retroperitoneum, normally, it should be feasible to detach it cleanly with minimal force and minimal bleeding. However, in this case, possibly due to concomitant fibrosis, a relatively strong force was necessary, and bleeding was more frequent than usual. However, ultimately, we were able to secure a 10 cm anal-side margin of the lesion. A small white nodule was found in the greater omentum, and intraoperative frozen section analysis revealed that it was a metastasis from adenocarcinoma. The operative time was 229 minutes, with a blood loss of 190 ml. A postoperative histopathological examination showed cecal cancer: SS, moderately differentiated adenocarcinoma, pN1b (2/16). The final diagnosis was T3N1bM1c Stage ⅣC. Serum IgG4 levels were not measured at any point after surgery.

For six months after surgery, capecitabine, oxaliplatin, and bevacizumab were administered. At the six-month mark, PET/CT showed no evidence of recurrent lesions, but due to the severe peripheral neuropathy of Common Terminology Criteria for Adverse Events (CTCAE) grade 3 [8], a decision was made to continue with capecitabine monotherapy because intraoperative findings revealed peritoneal dissemination, indicating Stage IV disease with a high risk of recurrence after discussion between the attending physician and the patient. At one year and four months postoperatively, the development of severe hand-foot syndrome (CTCAE grade 3) led to a temporary cessation of the drug therapy. Two months later, PET/CT revealed peritoneal dissemination recurrence, prompting the resumption of capecitabine, but the patient succumbed to cancer-related mortality the following year.

Immunohistochemical staining of the resected colon cancer specimens of both cases indicated that the circumferential stroma of the cancer gland was strongly positive for IgG and that the same area was also strongly positive for IgG4 (Figure 4). IgG4 was higher around the cancer tissue in a pathological examination.

**Figure 6:**
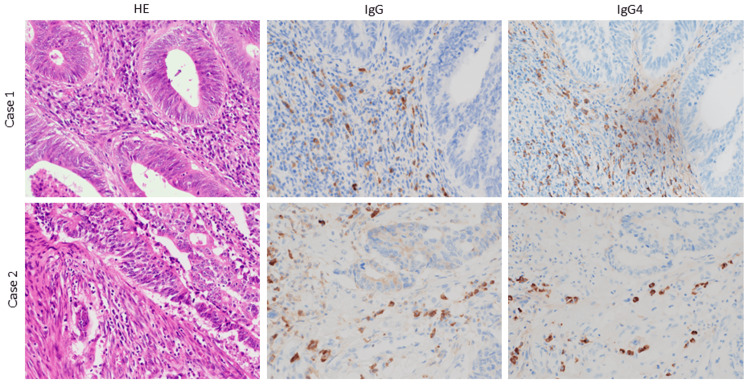
Immunohistochemistry The immunohistochemistry of the colon cancer specimen in both cases shows that the circumferential stroma of the cancer gland is strongly positive for IgG. The same area is also strongly positive for IgG4 staining. IgG: immunoglobulin G; IgG4: immunoglobulin G4; HE: hematoxylin and eosin

## Discussion

IgG4RD is most common in Asia and is listed as an intractable disease number 300 by the Japan Intractable Diseases Information Center [9]. It is estimated that there are 10,000 to 20,000 patients with IgG4RD in Japan. IgG4RD is thought to be an autoimmune disease, and steroid treatment is the first choice [10]. The following three criteria are required for a definitive diagnosis: clinical confirmation of growth, mass, nodule, or thickened lesion; a high serum IgG4 level (≥135 mg/dL); and confirmation of lymphocytes and plasma cells by histopathological examination. Both of our cases met only the first two criteria for IgG4RD, and thus, they were defined as possible cases [1].

The two cases of colorectal cancer complicated by IgG4RD reported here occurred within a short period, which provided us with two interesting perspectives: an oncological perspective on the relationship between IgG4RD and cancer development and a perspective on the characteristics of colorectal surgery complicated by IgG4RD. Yamamoto et al. reported that patients with IgG4RD are 3.5 times more likely to have a malignant disease than those without IgG4RD [11]. Regarding the sites of malignancy, Wallace et al. found that 20 (16%) of 125 IgG4RD cases were associated with malignancy, including seven cases of prostate cancer, four of malignant lymphoma, and two of colorectal cancer [12]. Several inferences have been made regarding the mechanism from an immunological perspective. In the cancer microenvironment of mice, Wang et al. found that IgG4 inhibits anti-cancer responses, potentially promoting cancer growth indirectly [13]. In 2021, Jordakieva et al. found that absolute serum IgG4 levels were higher in patients with colorectal cancer than in healthy persons and suggested that high IgG4 may work with macrophages in the microenvironment to suppress immunity and reduce the function of anti-cancer cells; as a result, colorectal cancer may occur in high IgG4 [14]. These findings suggest that patients with IgG4RD may be more susceptible to the development of some form of cancer. However, Hirano et al. found that the incidence of malignant tumors in patients with IgG4RD does not differ significantly from that in the general population, and thus, this issue remains controversial at present [15].

Another important point is that almost all cases of IgG4RD have a thickened retroperitoneum and multiple masses in the retroperitoneum that resemble enlarged lymph nodes. Thus, when performing surgery on a patient with IgG4RD, it is more likely to be difficult to remove the intestinal tract from the retroperitoneum, including both the tumor and non-tumor areas, because of fibrosis of the retroperitoneum. There have been few studies on surgery involving retroperitoneal dissection of colorectal cancer coexisting with IgG4RD. Our experience in the two cases reported here showed that dissection from the retroperitoneum of the colon was more difficult than usual. Mori et al. [16] also found strong adhesions around the duodenum, but other dissection procedures were not problematic. Therefore, while there is no consensus on retroperitoneal dissection, surgery should be approached with the understanding that strong adhesions may be present, depending on the case or local site. If surgeons are familiar with laparoscopy, it is acceptable to start the surgery with a laparoscopic approach. However, if adhesions are unexpectedly strong, conversion from laparoscopy to open surgery should be considered.

Imaging examinations such as PET/CT can also provide important information. In Case 1, PET/CT was useful in detecting new colorectal cancer lesions, whereas in Case 2, PET/CT was useful for the diagnosis of recurrent lesions. In recent years, PET/CT has generally been considered effective for assessing the lesion of IgG4RD, as well as for evaluating treatment response and monitoring [5,6]. It could be considered a valuable device for follow-up with patients with IgG4RD.

With regard to serum IgG4, Wallace et al. have shown that high serum IgG4 levels are associated with a more severe phenotype of IgG4RD [17], but we could not find any studies that clearly discuss the relationship between serum IgG4 and cancer. In Case 1, the high level of IgG4 contributed to the discovery of cancer, but this was considered to be incidental: IgG4 increased after reducing the dose of steroids, continued to rise after removal of the cancer, and decreased after administration of high-dose steroids (Figure 1). In Case 2, there was no correlation between the timing of cancer exacerbation and serum IgG4 levels. Therefore, based on these cases, there seems to be little relationship between serum IgG4 and carcinogenesis. Thus, at present, it remains unclear whether serum IgG4 functions as a tumor marker when considering the integration of previous literature and our findings. Future investigations comparing patients with colorectal cancer who have concomitant IgG4RD and those without may be necessary to elucidate this problem.

## Conclusions

During the follow-up of IgG4RD, we experienced two cases in which colorectal cancer was detected and subsequently underwent surgery. IgG4RD is known to be associated with several malignant diseases, although not commonly but rather infrequently, including colorectal cancer, and individuals with IgG4RD are believed to be more prone to malignancies compared to those without IgG4RD. However, the underlying mechanism remains incompletely elucidated. Serum IgG4 levels are not necessarily indicative of malignant diseases. In the present two cases, the utility of CT, PET/CT, and endoscopic examinations was highly evident, suggesting their potential effectiveness even in patients with IgG4RD. Histologically, enlarged lymph nodes and thickened retroperitoneum are frequently observed, necessitating differentiation between changes associated with malignancies and those without. Therefore, during surgery, planning incorporating considerations for adhesions when removing organs from the retroperitoneum was deemed crucial. In other words, while laparoscopy may be feasible in some cases, in others where laparoscopy is challenging, open surgery may be preferable.
